# An integrated study combining network toxicology machine learning and molecular simulation reveals the molecular mechanisms of permanent hair dyes in breast cancer

**DOI:** 10.1007/s12672-026-04585-1

**Published:** 2026-02-08

**Authors:** Xiaolu Yang, Yilun Li, Tianqi Zhang, Binglu He, Jingyan Wang, Shiyu Zhang, Li Ma

**Affiliations:** 1 Department of Breast Disease Center, The Fourth Hospital of Hebei Medical University, Shijiazhuang, Hebei China; 2 Hebei Medical University, Shijiazhuang, China; 3 Tangshan Gongren Hospital, Tangshan, China; 4 Xingtai People’s Hospital, Xingtai, China

**Keywords:** Permanent hair dyes, BC, Network toxicology, Molecular docking, Molecular dynamics simulation

## Abstract

Permanent hair dyes have been linked to an increased risk of breast cancer (BC), though the underlying mechanisms remain unclear. To address this knowledge gap, our investigation employed an integrated approach combining network toxicology, molecular docking, molecular dynamics simulations, and machine learning to decipher the molecular mechanisms by which permanent hair dyes might promote BC pathogenesis. Five permanent hair dye ingredients classified by IARC as carcinogenic were included in this study: p-phenylenediamine, resorcinol, pyridine, Disperse Yellow 3, and HC Blue No. 2. These chemicals can regulate BC progression through various signaling pathways, with key core targets identified as HSP90AA1, HSP90AB1, ESR1, CDK1, STAT3, MAPK8, HDAC1, and SRC. A machine learning model comprising 128 algorithms confirmed that these eight targets possess strong prognostic predictive capabilities for BC. Subsequent SHAP analysis revealed SRC, HSP90AB1, HSP90AA1 and CDK1 as the key contributors to prognostic prediction, with each being highly expressed in BC and linked to poor clinical prognosis. Notably, among all chemicals screened, Disperse Yellow 3 exhibited the strongest binding affinity to these four key targets, demonstrating the strongest association with BC risk.

**Supplementary Information:**

The online version contains supplementary material available at 10.1007/s12672-026-04585-1.

## Introduction

Individuals are exposed to a variety of chemicals in daily life, many of which are carcinogenic. However, a large amount of harmful substances are hidden in daily necessities, and users are often unaware of them. Permanent hair dye is a typical example—this common product contains a variety of carcinogens [1, 2]. Permanent hair dyes, also known as oxidative hair dyes, rely on an oxidation process for coloring. Typically, they consist of three components: (1) intermediate agents like p-phenylenediamine (PPD); (2) coupling agents such as resorcinol (REN) and m-phenylenediamine; and (3) oxidizing agents like hydrogen peroxide. The intermediate and coupling agents together form dye precursors. During application, these precursors undergo oxidation in the presence of an oxidizing agent, resulting in the formation of colored macromolecules that are encapsulated within the hair, thereby altering its color [3]. Variations in the types and proportions of intermediates and coupling agents produce different shades.

More than 30% of women in Western countries use hair dyes [4], and with its widespread use, concerns about its potential health effects are growing. Some studies show that the use of permanent hair dye may be associated with breast cancer (BC), the most prevalent cancer in women globally [5]. Research shows that women who use hair dyes have a 23% higher risk of BC than non-users [6]. In addition, a prospective cohort study revealed that the use of permanent hair dyes increases the risk of BC in black women by 45% and white women by 7% [7].

Although epidemiological studies have confirmed that permanent hair dyes are associated with an increased risk of BC, their potential mechanism remains unclear. The progress in the field of toxicology, especially the development of network toxicology, enables researchers to comprehensively analyse the impact of chemicals on the human body from a holistic perspective [8]. However, applying network toxicology alone can only identify the targets at which compounds act on diseases, without further determining the impact of these targets on disease prognosis. To better assess the impact of compounds on diseases and even disease prognosis, we present for the first time an integrated computational framework that combines network toxicology, machine learning, molecular docking, and molecular dynamics simulations. This integrated approach not only identifies compounds' potential core targets and signaling pathways in diseases but also validates their prognostic relevance in disease contexts. Overall, this multidisciplinary methodology will yield new insights into the safety of permanent hair dyes.

## Methods

### Identification of targets for carcinogenic chemicals in permanent hair dyes

First, we identified the chemical components contained in permanent hair dyes through a comprehensive literature review and evaluated their toxicity using the PubChem database. All compounds classified as carcinogens in the IARC registry were included in the analysis. Ultimately, five compounds were selected for further study: PPD, REN, pyridine (PYD), Disperse Yellow 3 (DY3), and HC Blue No. 2 (HB2). These chemicals are summarized in Table 1. Potential targets for these chemicals were retrieved from three databases: SEA (https://sea.bkslab.org/), STP (http://swisstargetprediction.ch/), and STITCH (http://stitch.embl.de/) [9]. All three databases provide target information for compounds.

**Table 1:** Carcinogenic chemicals in permanent hair dyes

Chemical	Molecular formula	Molecular weight	Structure
p-phenylenediamine	C_6_H_8_N_2_	108.14	
Resorcinol	C_6_H_6_O_2_	110.11	
Pyridine	C_5_H_5_N	79.10	
Disperse Yellow 3	C_15_H_15_N_3_O_2_	269.3	
HC Blue No. 2	C_12_H_19_N_3_O_5_	285.3	

### Confirmation of a common target for permanent hair dyes and BC

To identify BC-related targets, the Genecards (with a relevance score of 10 or more) (https://www.genecards.org/), TTD (https://db.idrblab.net/ttd/), and OMIM (https://www.omim.org/) databases were searched using the keyword "breast cancer" [10]. The VennDiagram package in R (version 4.2.1) was used to find overlapping targets between permanent hair dye chemicals and BC.

### Enrichment analysis

Gene Ontology (GO) and Kyoto Encyclopedia of Genes and Genomes (KEGG) enrichment analyses were performed on the intersecting targets to explore the biological implications of permanent hair dyes in BC.

### Construction of the protein–protein interaction (PPI) network

A PPI network for the intersecting targets was constructed using the STRING database with a minimum interaction score of 0.9, visualized with Cytoscape (version 3.9.1).

### Identification of hub targets

The network analysis tool in Cytoscape software was used to analyze the topological parameters of the PPI network. Targets with degree and betweenness centrality values greater than twice the mean were considered potential core targets, resulting in the identification of eight such targets. To evaluate the prognostic value of the eight core targets, we constructed 128 ensemble models using 10 machine learning algorithms (Supplementary Table S1), trained them on the GSE20685 dataset, and validated them on two independent cohorts (GSE16446 and GSE48390), with the predictive performance assessed by the average AUC value. Ultimately, the three datasets were merged and normalized to eliminate batch effects, followed by a SHapley Additive exPlanations (SHAP) analysis to quantify the contribution of each target. Additionally, we further analyzed the differential expression of these core targets in BC and normal breast tissue, as well as their association with prognosis, using the TCGA-BRCA cohort.

### Single-cell analysis

Four targets were identified as key targets closely associated with BC prognosis: HSP90AA1, HSP90AB1, CDK1, and SRC. We analyzed the expression levels of these four targets in different cell types within BC using the single-cell sequencing dataset GSE161529.

### Molecular docking analysis

Molecular docking was performed to predict the binding affinities between the five hair dye constituents and the four core protein targets [11]. The three-dimensional crystal structures of the target proteins—HSP90AA1 (PDB ID: 1BYQ), HSP90AB1 (PDB ID: 1QZ2), CDK1 (PDB ID: 4Y72), and SRC (PDB ID: 1A07)—were retrieved from the Protein Data Bank (https://www.rcsb.org/). Protein structures were preprocessed by removing water molecules and adding hydrogen atoms using PyMOL. The SDF files of the chemical ligands were obtained from PubChem and subsequently subjected to energy minimization using Chem3D [12]. All docking simulations were carried out using AutoDockTools (version 1.5.7) with parameters detailed in Supplementary Table S2. To validate the reliability of our docking protocol, we performed a redocking procedure. The native ligand was re-docked into its original active site using the parameters described above. The root-mean-square deviation (RMSD) between the redocked pose and the original crystallographic pose was calculated for each protein. All RMSD values were below 2.0 Å, confirming the accuracy of our docking workflow (Supplementary Table S3). Following this validation, molecular docking of the five hair dye chemicals was conducted. The resulting protein–ligand complexes were visualized, and their binding energies were calculated using PyMOL.

### Molecular dynamics simulation

Molecular dynamics simulations were carried out with Gromacs 2023 for 100 ns at 300 K and 1 bar pressure. The CHARMM 36 force field parameters were applied to the proteins, while ligand topologies were generated using GAFF2 [13]. Electrostatic interactions were modelled with particle mesh Ewald and Verlet algorithms, and a 1.0 nm cutoff was used for van der Waals and Coulomb interactions.

## Results

### Identifying potential targets for permanent hair dyes and BC

**Fig. 1:**
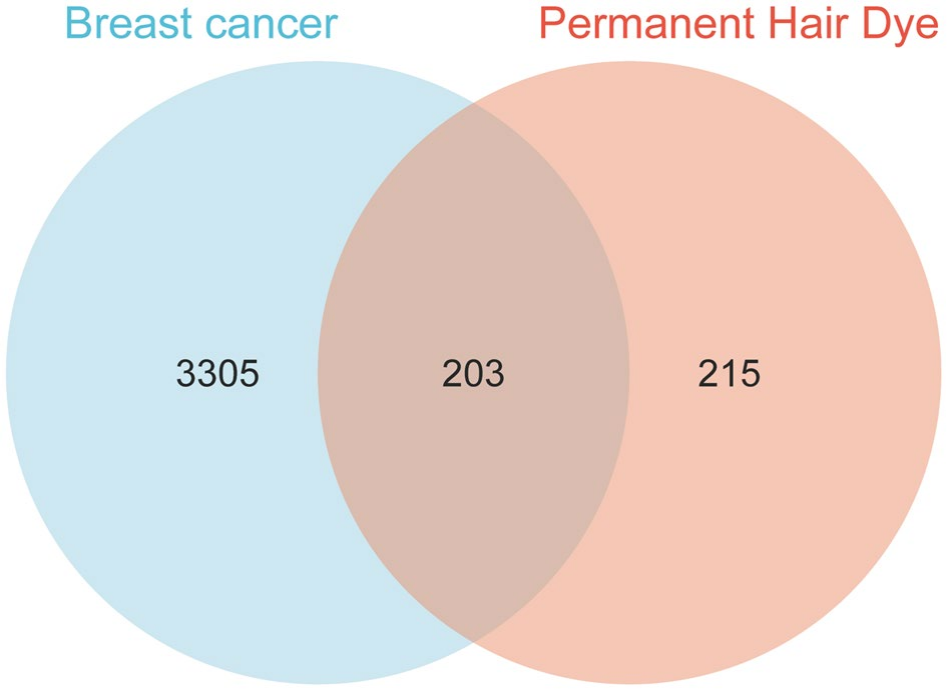
Common targets associated with permanent hair dyes and breast cancer

After removing duplicate targets, a total of 418 targets for the five chemical components of permanent hair dyes were identified from the SEA, STP, and STITCH databases. Additionally, 3,508 BC-related targets were retrieved from the Genecards, TTD, and OMIM databases. Integration of these targets resulted in 203 intersecting targets, considered potential mediators of the carcinogenic effects of permanent hair dyes on BC (Fig. 1). A complete list of the targets for permanent hair dyes, BC, and their intersections is available in Supplementary Table S4.

### Constructing a network of permanent hair dyes and potential targets

**Fig. 2:**
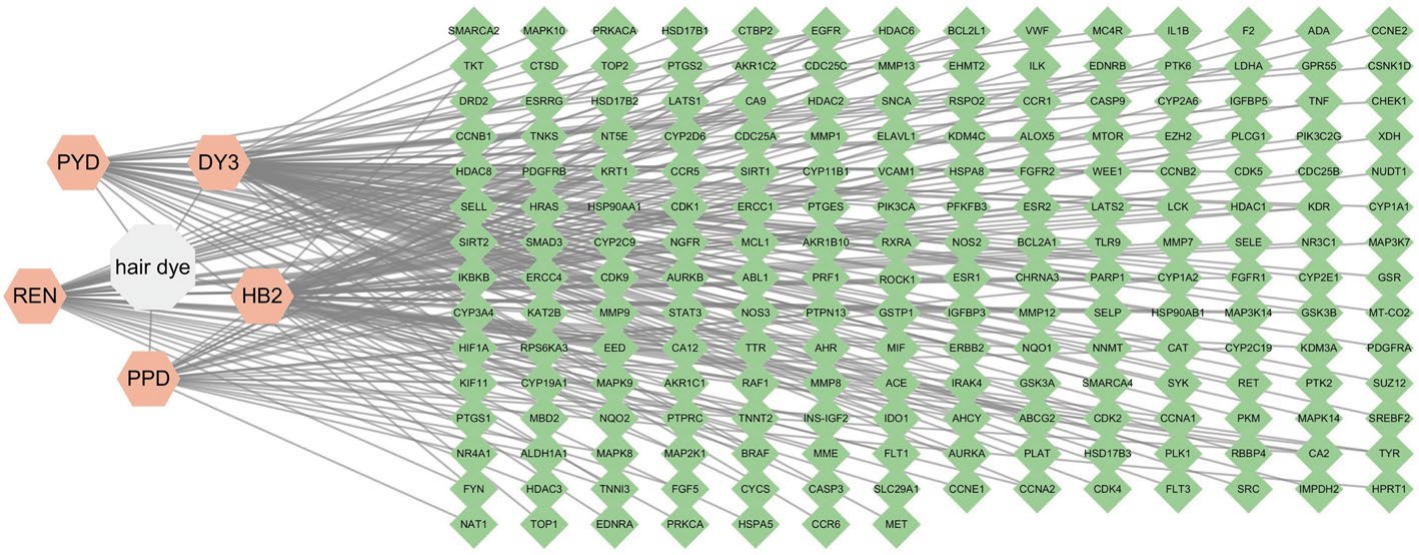
Relationship between permanent hair dyes and their corresponding targets

To investigate the impact of permanent hair dyes on BC, a network was constructed linking the five carcinogenic chemicals to the 203 intersecting genes (Fig. 2). Among the five chemicals, DY3 exhibited the strongest association with BC, targeting 79 genes, followed by HB2 (68), REN (46), PYD (34), and PPD (29).

### Enrichment analyses

**Fig. 3:**
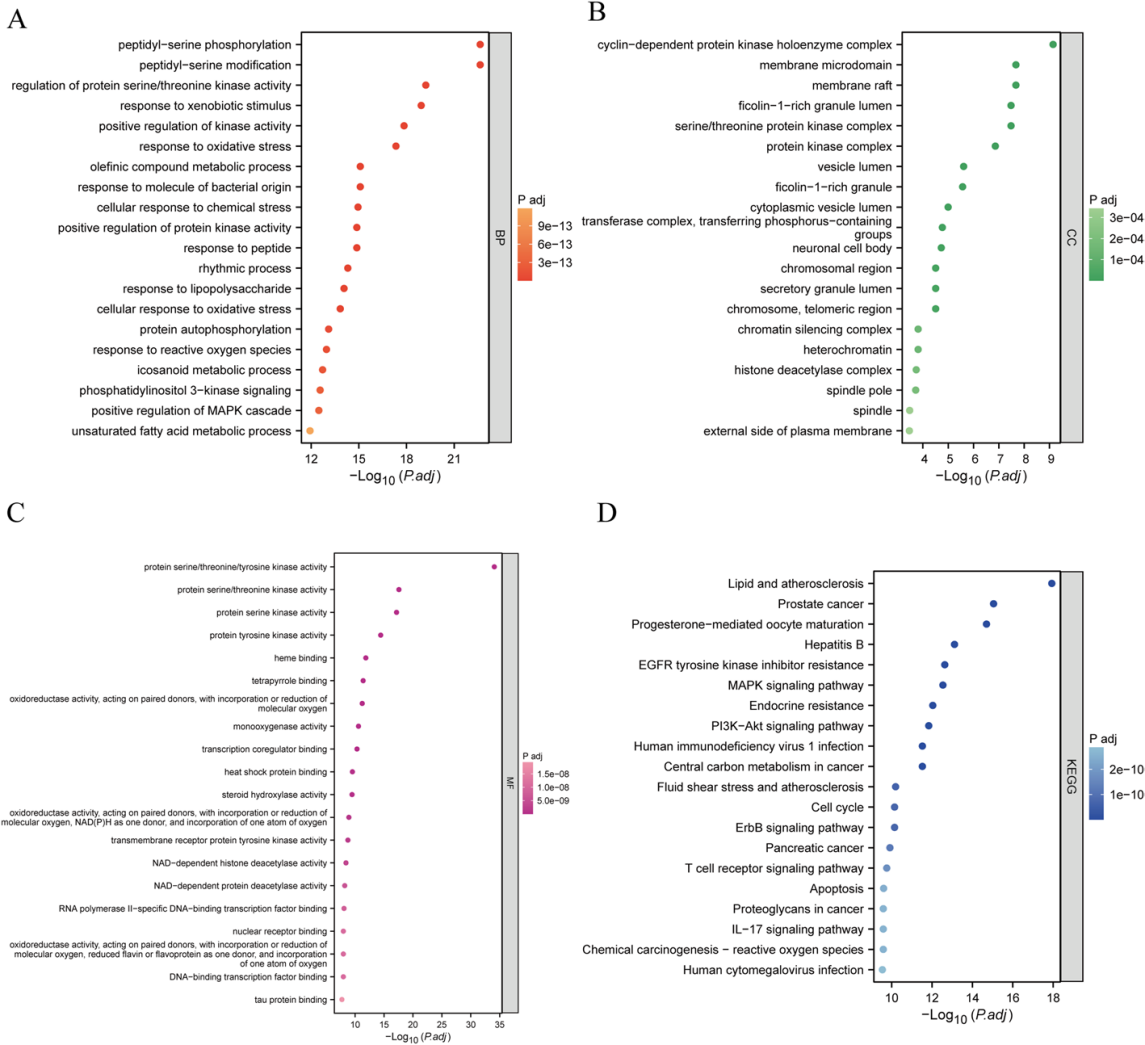
Enrichment analysis of 203 common targets. **A–C** Top 20 items in **A** BP, **B** CC, and **C** MF affected by permanent hair dyes in BC. **D** Top 20 KEGG pathways influenced by permanent hair dyes in BC

GO and KEGG analyses were conducted to explore the functions and pathways influenced by these chemicals. A total of 2789 GO terms were identified, including 2488 biological processes (BPs), 92 cellular components (CCs), and 209 molecular functions (MFs) (Supplementary Table S5). The top 20 GO terms are visualized in Fig. 3A–3C. Furthermore, the five chemicals affected 168 KEGG pathways, with several cancer-related pathways among the top 20, such as MAPK signaling, PI3K-Akt signaling, the cell cycle, and apoptosis (Fig. 3D). Eighteen of these pathways are directly linked to cancer, including those related to prostate, pancreatic, breast, and thyroid cancers (Supplementary Table S5).

### Building the PPI networks and identifying potential core targets

**Fig. 4:**
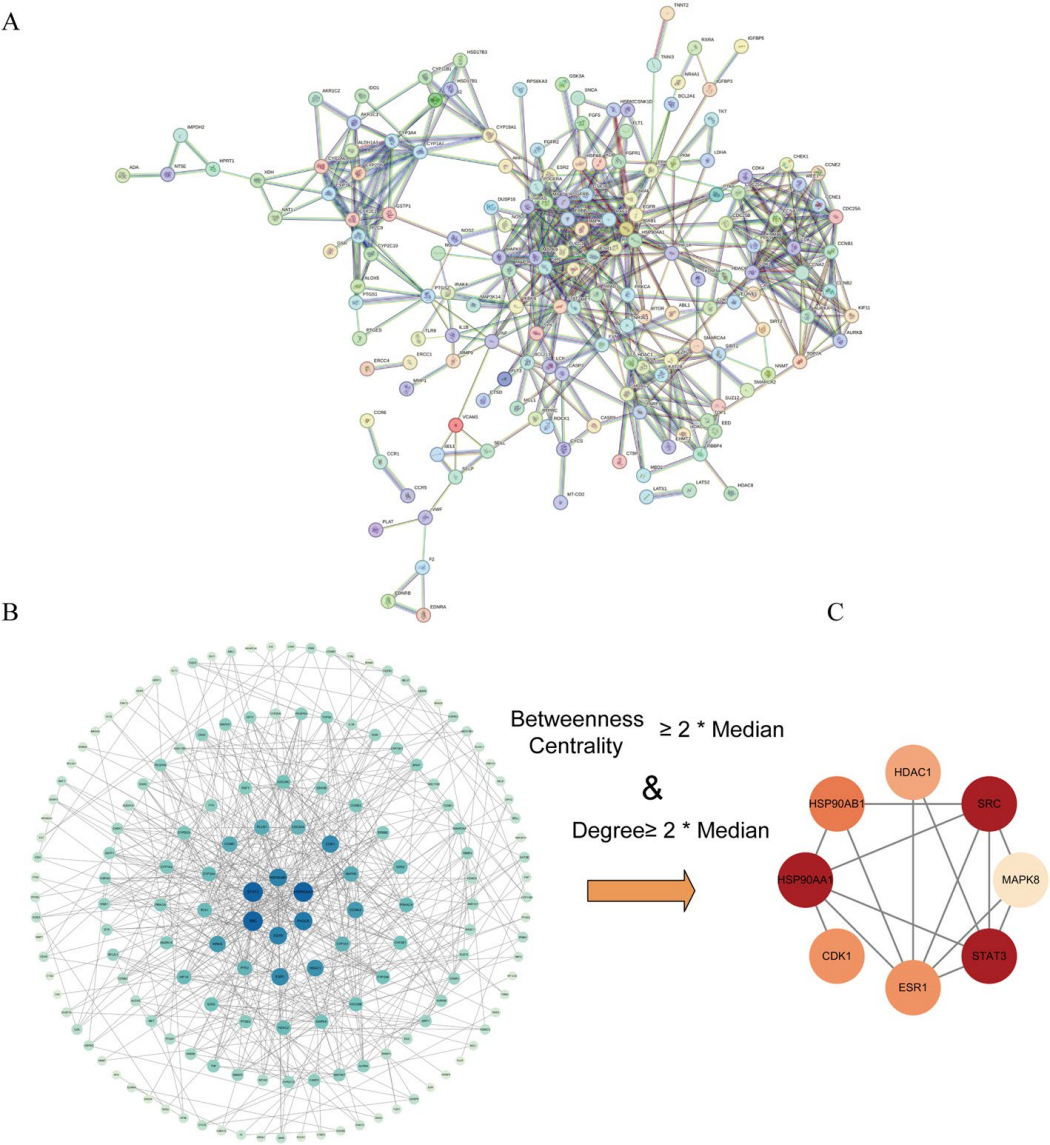
PPI network and identification of potential core targets for 203 common targets. **A** PPI network for 203 common targets. **B** Processed PPI network visualized using Cytoscape software. **C** Potential core targets obtained by screening

To elucidate the mechanisms by which these chemicals contribute to BC, a PPI network was generated using the STRING database for the 203 intersecting genes (Fig. 4A) and visualized in Cytoscape (Fig. 4B). Larger nodes and darker colors indicate higher degree values. Core targets were identified by analyzing the topological parameters of the PPI network, including degree and betweenness centrality, both indicative of node importance [14]. The average degree value was 6.80, and the average betweenness centrality was 0.12. Eight targets with both degree and betweenness centrality values greater than twice the mean were selected as potential core targets: HSP90AA1, HSP90AB1, ESR1, CDK1, STAT3, MAPK8, HDAC1, and SRC (Fig. 4C).

### Machine learning identifies 4 key targets closely associated with BC prognosis

**Fig. 5:**
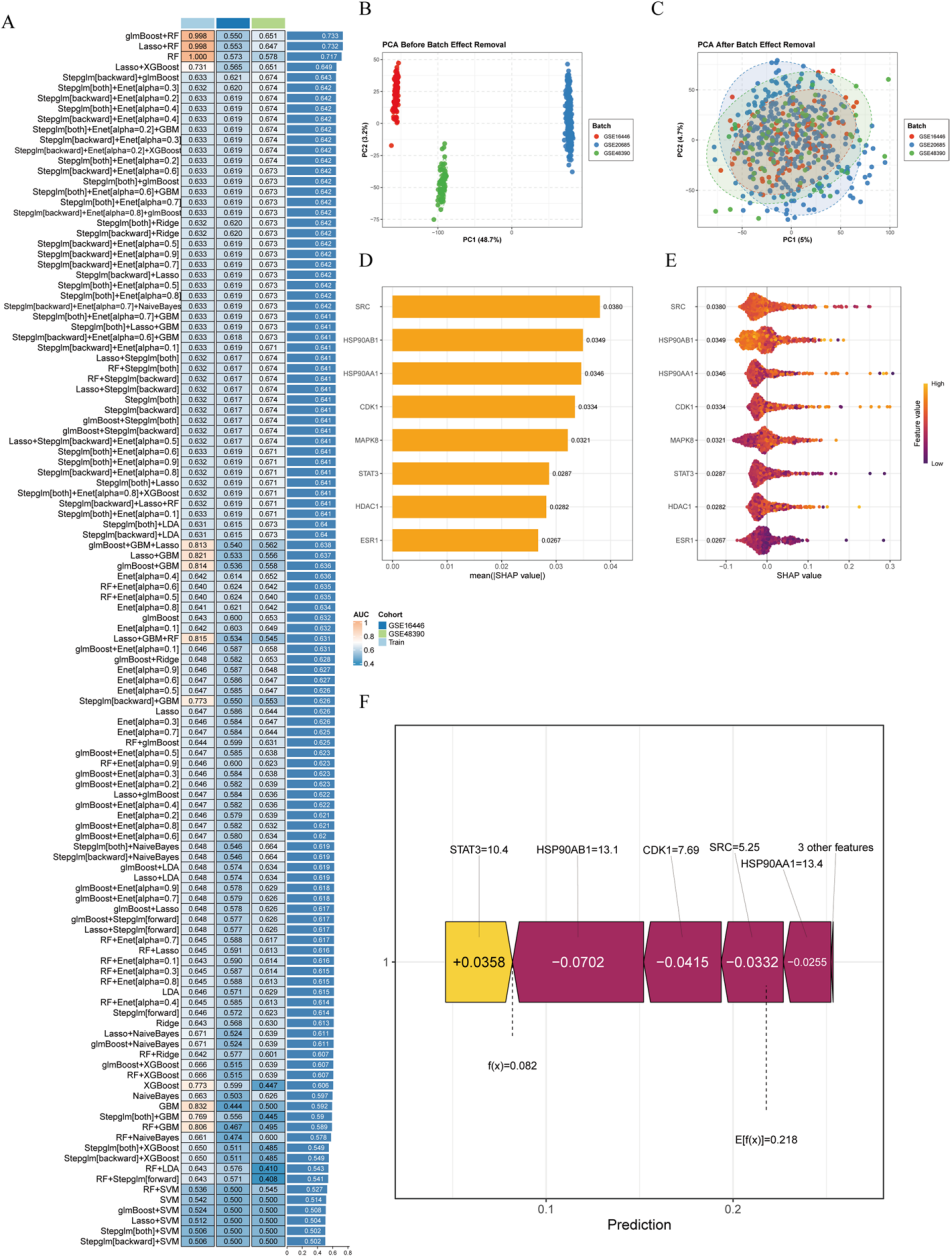
Identification of core prognostic genes in breast cancer (BC) by integrating machine learning and SHAP analysis. **A** Performance of machine learning algorithms in predicting prognosis, quantified by mean AUC values. **B**–**C** PCA scatter plots of the dataset (**B**) before and (**C**) after normalization, illustrating the effect of normalization in mitigating batch effects. **D, E** SHAP analysis evaluating the contribution of the eight core features, represented as a (**D**) bar chart and an (**E**) beeswarm plot. **F** SHAP summary plot depicting feature-wise impact on model predictions

To comprehensively assess the relationship between eight potential core targets and prognosis, we constructed 128 machine learning models. As illustrated in Fig. 5A, models based on these targets effectively predicted patient outcomes. The ensemble model combining glmBoost and Random Forest (RF) demonstrated the best performance, achieving an AUC of 0.733. Next, we merged these three datasets and normalized the gene expression matrix to eliminate batch effects. Principal component analysis (PCA) indicates that the batch effects in the three datasets have been effectively reduced (Fig. 5B–C). We quantified the contribution of these eight targets to the model using SHAP analysis. The top four targets with the highest contributions were SRC, HSP90AB1, HSP90AA1 and CDK1 (Fig. 5D–E). Force-directed analysis further demonstrates that these four targets are primary negative regulators of the shap value, indicating a negative correlation with prognosis in patients with BC (Fig. 5F).

**Fig. 6:**
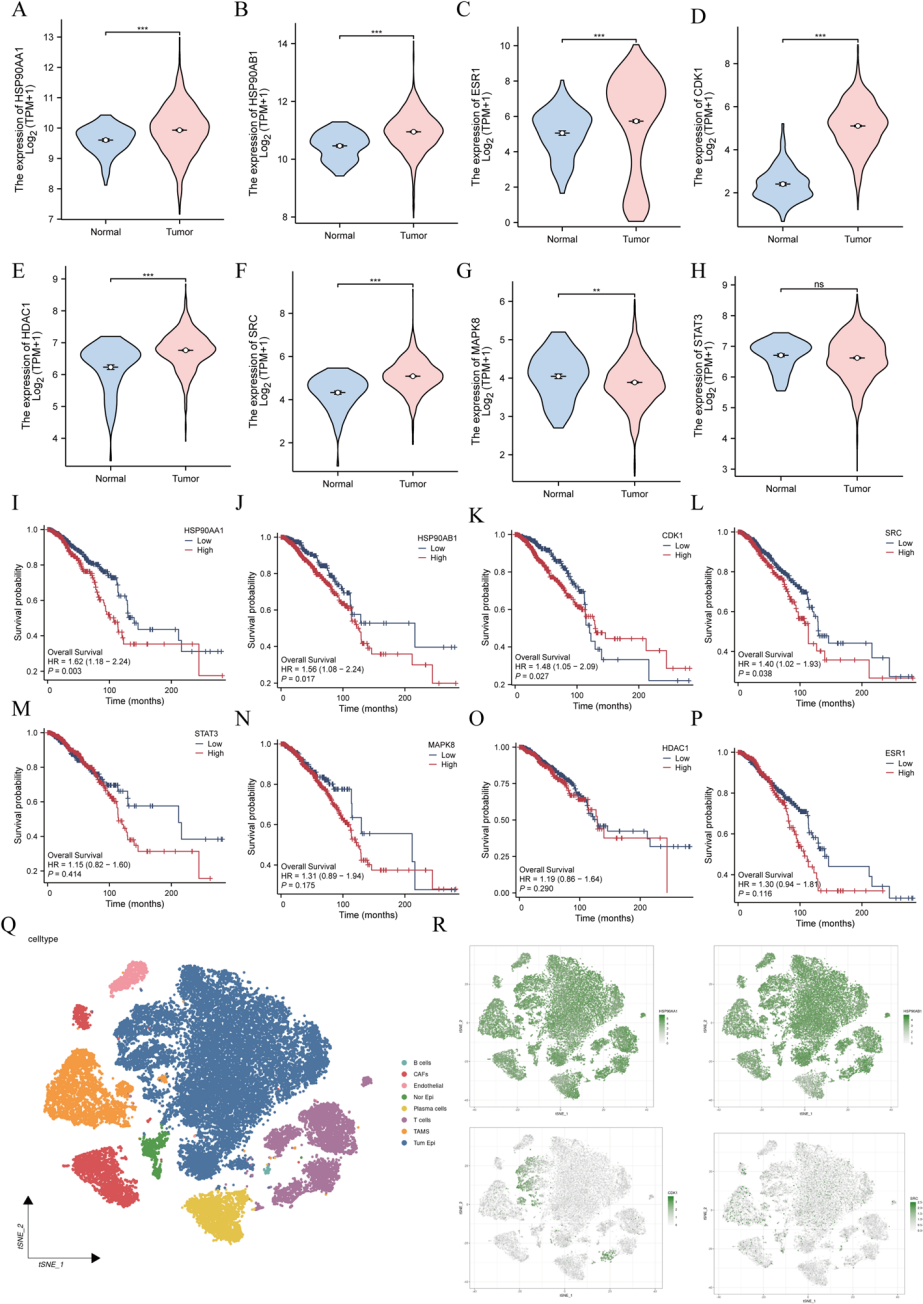
Core gene expression, prognostic correlation, and cellular profiling in BC. **A–H** Expression levels of eight targets—**A** HSP90AA1, **B** HSP90AB1, **C** ESR1, **D** CDK1, **E** HDAC1, **F** SRC, **G** MAPK8, and **H** STAT3—in BC samples from the TCGA-BRCA cohort. **I–P** Prognostic associations of gene expression for **I** HSP90AA1, **J** HSP90AB1, **K** CDK1, **L** SRC, **M** STAT3, **N** MAPK8, **O** HDAC1, and **P** ESR1 in the TCGA-BRCA cohort. **Q** The cellular landscape of BC in the GSE161529 cohort. **R** The expression profiles of four core targets in BC. (**P < 0.01; ***P < 0.001; ns: no significance)

Additionally, we further utilized the TCGA-BRCA cohort to explore the relationship between these targets and BC. Among the eight core targets, the expression levels of HSP90AA1, HSP90AB1, ESR1, CDK1, HDAC1 and SRC in BC tissue were significantly higher than those of normal breast tissue (Fig. 6A–6F). On the contrary, the expression level of MAPK8 in BC tissue decreased (Fig. 6G). In addition, there is no significant difference in the expression of STAT3 in BC tissue and normal tissue (Fig. 6H). Regarding the analysis of prognostic associations, we found that the high expression levels of HSP90AA1, HSP90AB1, CDK1 and SRC were negatively correlated with the overall survival of patients with BC (Fig. 6I–6L), while the expression levels of the remaining four core targets were not related to the survival rate of BC (Fig. 6M–6P). The finding is consistent with the results of machine learning and SHAP analysis, which further confirms the correlation between these four key targets (HSP90AA1, HSP90AB1, CDK1 and SRC) and the poor prognosis of BC.

### Expression profile of core targets in BC

The cellular landscape of the BC microenvironment is depicted in Fig. 6Q. Corresponding expression profiling of the four core targets (Fig. 6R) revealed that HSP90AA1 and HSP90AB1 were widely expressed across both epithelial and mesenchymal cancer cells. In contrast, CDK1 expression was predominantly localized to epithelial cells and T cells, while SRC was mainly detected in epithelial cells and tumor-associated macrophages.

### Molecular docking analysis

**Fig. 7:**
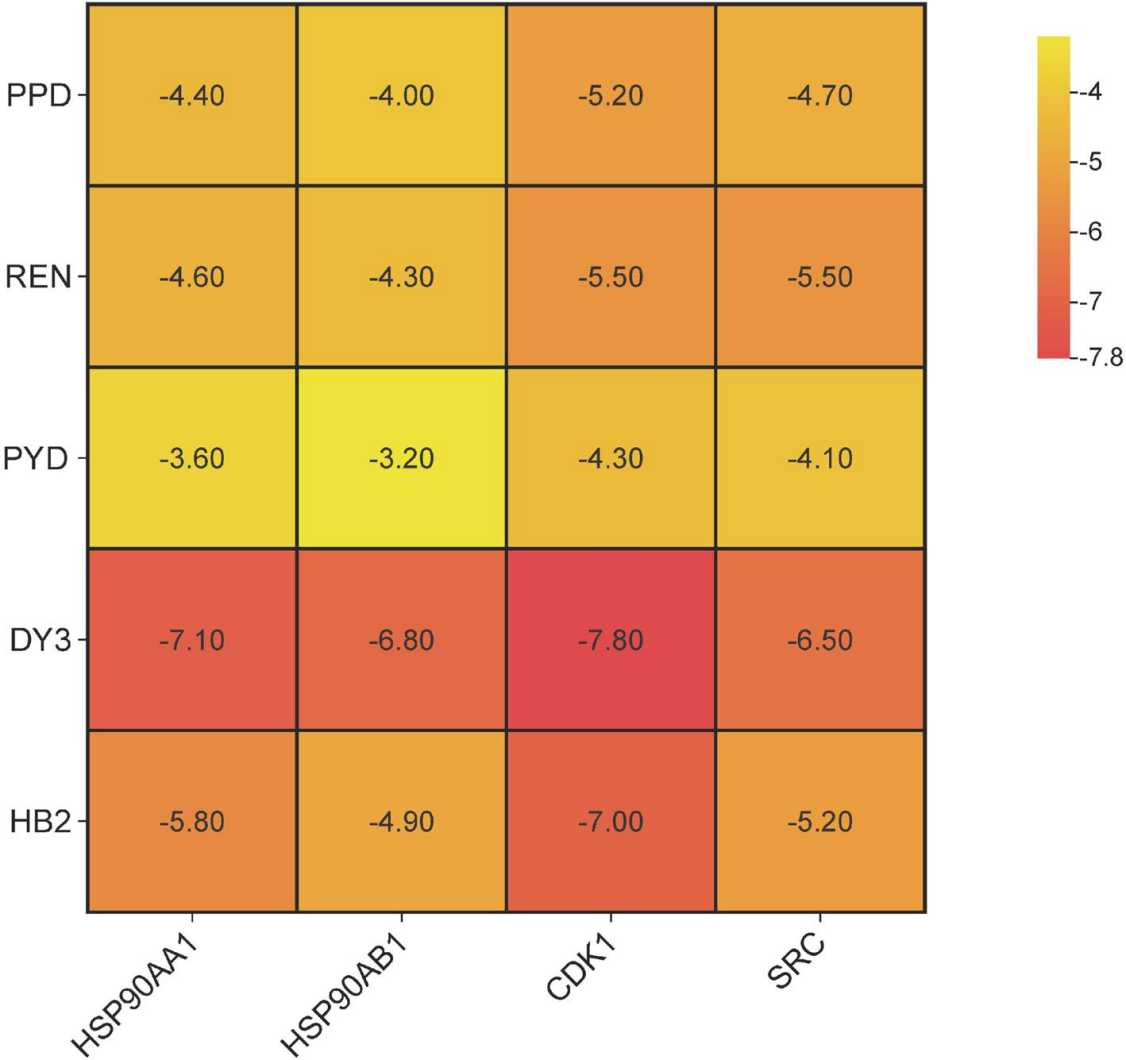
The binding energy of molecular docking between five chemicals and four core targets

**Fig. 8:**
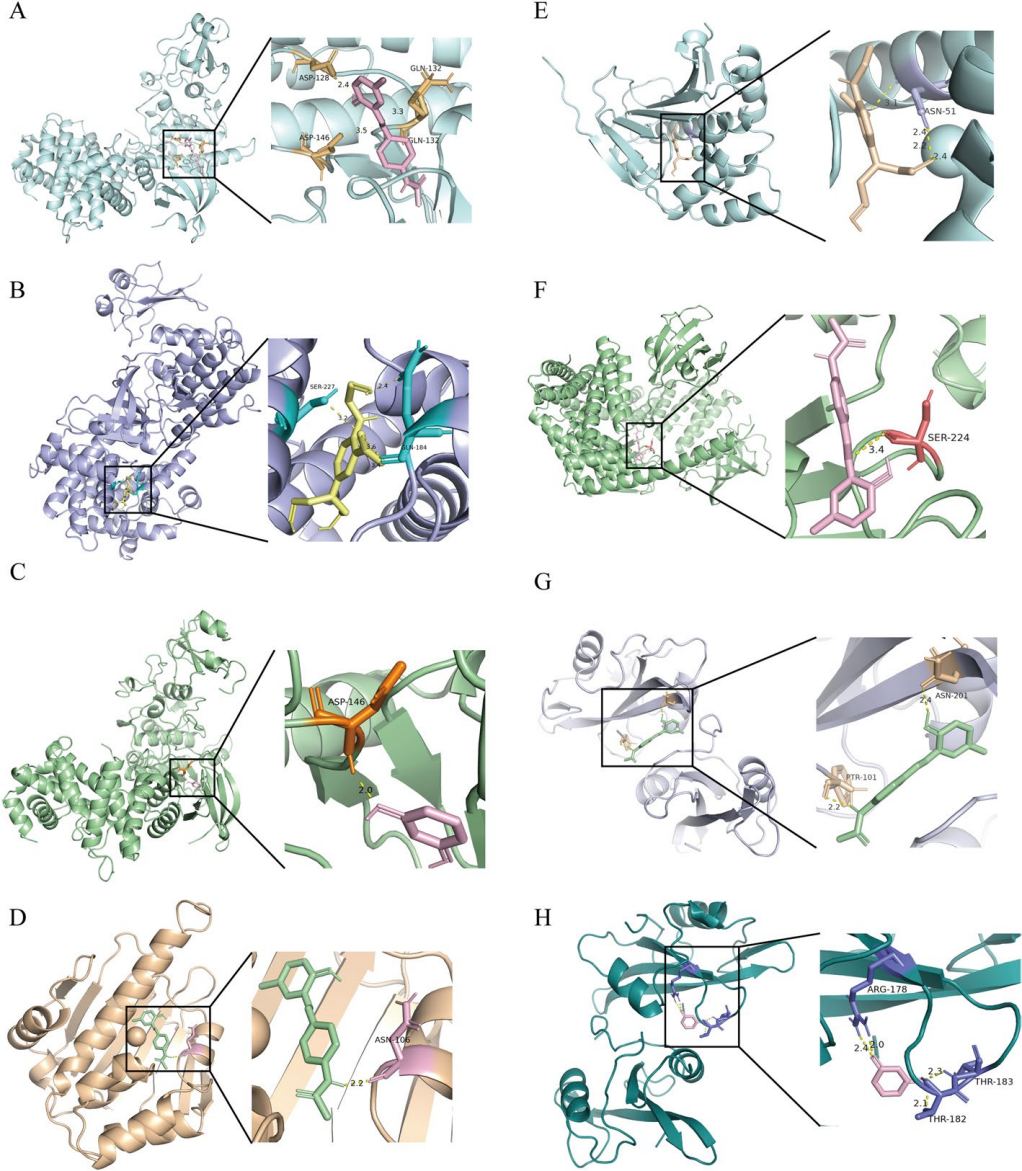
Molecular docking results of chemicals binding to targets. **A–H** Molecular docking plots: **A** CDK1-DY3, **B** CDK1-HB2, **C** CDK1-REN, **D** HSP90AA1-DY3, **E** HSP90AA1-HB2, **F** HSP90AB1-DY3, **G** SRC-DY3, and **H** SRC-REN

Through bioinformatics analysis, we identified HSP90AA, HSP90AB1, CDK1, and SRC as core targets, as they are highly expressed in BC and closely associated with poor prognosis. To investigate the relationship between these four core targets and five chemical compounds, we performed molecular docking between the targets and the compounds and calculated their binding energies. Figure 7 displays the binding energies of these interactions, with values below -5.5 indicating strong binding affinity between the target and compound [15]. Strong binding was observed for CDK1-DY3, HSP90AA1-DY3, CDK1-HB2, HSP90AB1-DY3, SRC-DY3, HSP90AA1-HB2, CDK1-REN, and SRC-REN. Notably, DY3 showed strong binding affinity with all four core targets. Figure 8 displays visualized molecular docking images, revealing hydrogen bonds formed in all eight complexes (Table 2). Crucially, we observed that the hydrogen bond-forming sites between the compound and the protein are located within the protein’s functional domains. This indicates that the compound can bind to the protein and exert its effects by influencing the protein's biological functions.

**Table 2:** Number and position of hydrogen bonds in molecular docking

Receptor-ligand complex	Number of hydrogen bonds	Hydrogen-bonding residues
CDK1-DY3	3	ASP-128, GLN-132, ASP-146
CDK1-HB2	4	GLN-184, SER-227
CDK1-REN	1	ASP-146
HSP90AA1-DY3	1	ASN-106
HSP90AA1-HB2	4	ASN-51
HSPP90AB1-DY3	1	SER-224
SRC-DY3	2	PTR-101, ASN-201
SRC-REN	4	ARG-178, THR-182, THR-183

### Molecular dynamics simulations

**Fig. 9:**
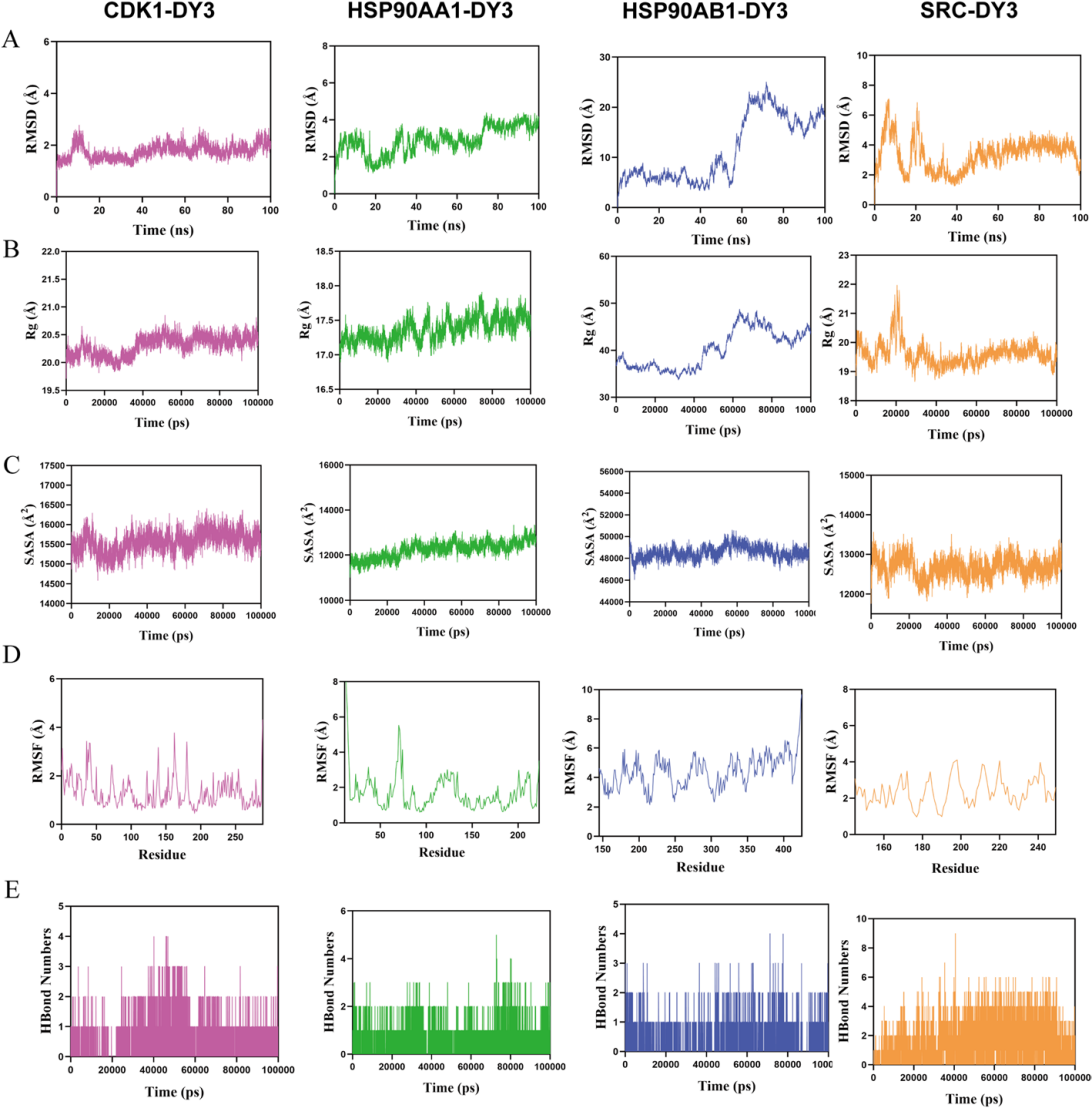
Molecular dynamics simulations of DY3 binding with four core targets. **A–E** Values for the four complexes: **A** RMSD, **B** Rg, **C** SASA, **D** RMSF and **E** Number of Hbonds

Molecular docking analysis indicated that DY3 has a strong binding ability with four core targets. In order to further explore its binding stability, we simulated the molecular dynamics of four complexes. The RMSD was used to evaluate the conformational stability of proteins and ligands. The smaller the deviation value, the higher the conformational stability of the protein–ligand complex. The structural changes of the protein–ligand complexes were evaluated by the radius of gyration (Rg). The smaller the Rg value, the more compact the structure. It was shown that the CDK1-DY3, HSP90AA1-DY3, and SRC-DY3 complexes quickly reached equilibrium during the simulation, with final RMSD values below 5 Å (Fig. 9A). Furthermore, the Rg values of these three complexes remained stable throughout the simulation, indicating that they were tightly packed and stably bound (Fig. 9B). In contrast, the RMSD and Rg values for HSP90AB1-DY3 fluctuated during the simulation. The solvent-accessible surface area (SASA) was used to evaluate protein folding and stability, and the SASA values for all four complexes remained stable during the simulations (Fig. 9C). Moreover, we employed the root mean square fluctuation (RMSF) metric to assess the flexibility of amino acid residues in proteins. The results revealed that the RMSF values for the complexes were predominantly below 5 Å, further corroborating the stability of the protein–ligand interactions (Fig. 9D). Additionally, hydrogen bonding plays a critical role in ligand–protein interactions. Figure 9E illustrates the number of hydrogen bonds between DY3 and the proteins during the simulations. Hydrogen bonds were consistently formed between DY3 and the four proteins, with at least two hydrogen bonds observed at most time points, suggesting stable interactions. Overall, the four complexes exhibited strong stability during molecular dynamics simulations, with the binding of DY3 to CDK1, HSP90AA1, and SRC being particularly stable.

## Discussion

In modern society, the use of hair dyes has become increasingly common, with individuals starting to color their hair at younger ages. This trend increases the possibility of long-term exposure to certain chemicals in hair dyes that have been proven to pose health risks [1]. Hair dyes are associated with a variety of health problems, including allergic reactions, hair loss, and respiratory disorders [16–19]. In addition, research shows that the use of permanent hair dyes is associated with an increased risk of a variety of cancers, including bladder cancer, hematopoietic cancer, and BC [20–22]. Despite the existence of these associations, the potential mechanism is still unclear. By integrating network toxicology, molecular docking, molecular dynamics simulation and bioinformatics technology, this study has preliminarily revealed the mechanism by which permanent hair dyes may induce BC.

Through a comprehensive literature search and screening, five carcinogenic chemicals commonly found in permanent hair dyes were identified: PPD, REN, PYD, DY3, and HB2. These chemicals are classified as carcinogens by IARC [23]. Network toxicology analyses indicated these chemicals may regulate the progression of BC through multiple signalling pathways, and their core targets include HSP90AA1, HSP90AB1, ESR1, CDK1, STAT3, MAPK8, HDAC1, and SRC. Further screening through bioinformatics analyses, among which HSP90AA1, HSP90AB1, CDK1 and SRC were identified as core targets due to their high expression in BC tissue and closely related to poor prognosis. Molecular docking and molecular dynamics simulations further confirmed that DY3 exhibits the highest binding affinity with the mentioned four targets, making it the compound most strongly associated with BC risk.

These four core targets all play important biological roles in the human body. Specifically, HSP90AA1 and HSP90AB1, as central components of the heat shock protein family, function as essential molecular chaperones that facilitate the folding, stability, and maturation of a wide range of client proteins—many of which are implicated in oncogenic signaling [24]. By coordinating multiple regulatory pathways, they help maintain proteostasis and regulate gene expression under physiological and stress conditions [25]. Interference with HSP90 function may lead to the ubiquitination and degradation of its client proteins, thereby disrupting key survival and proliferation pathways in breast tissue [26]. CDK1, as a key regulator of the G2/M transition in the cell cycle, belongs to the cyclin-dependent kinase family [27]. The potential impact of permanent hair dye components on CDK1 may induce cell cycle arrest at the G2/M checkpoint. In normal breast epithelial tissue, persistent cell cycle arrest—particularly within stem or progenitor cell populations—may promote genomic instability, thereby increasing cancer risk [28]. SRC, the first identified proto-oncogene in mammals and a non-receptor tyrosine kinase, serves as a signaling hub governing proliferation, adhesion, and survival [29]. Elevated or sustained SRC activation is widely recognized as a driver of tumor initiation and progression [30]. The strong binding affinity observed between permanent hair dye components and SRC raises the possibility of modulated kinase activity, which may alter downstream signaling cascades, further influencing breast cell fate.

Although our study predicts high-affinity binding between certain permanent hair dye components and carcinogenic targets, actual toxicity in practical applications depends on multiple factors, including dermal absorption, systemic distribution, and metabolic detoxification processes. Nevertheless, given the frequency and chronicity of hair dye use—often spanning decades—even low-level exposure could lead to bioaccumulation or sustained pathway modulation, meriting careful evaluation.

## Limitations

This study provides only preliminary insights into the potential mechanisms by which permanent hair dyes may induce BC, and several limitations remain. First, more epidemiological studies are needed to strengthen the link between exposure to these chemicals and BC incidence. Second, as this study is computational in nature, the predictive results obtained must be validated through experimental methods (such as in vitro binding assays and toxicokinetic verification) to confirm the toxicological effects of permanent hair dyes on humans.

## Conclusion

In conclusion, this study combines multiple approaches to investigate the effects of permanent hair dyes on BC. Five carcinogenic chemicals were identified, with DY3 showing the strongest association with BC risk. Four core targets—HSP90AA1, HSP90AB1, CDK1, and SRC—were found to be closely associated with BC. Molecular docking indicated that all five chemicals bind stably to these targets, with DY3 showing the most potent interaction.

## Supplementary Information

Supplementary Material 1.

Supplementary Material 2.

Supplementary Material 3.

Supplementary Material 4.

Supplementary Material 5.

## Data Availability

All data mentioned in this paper are provided in the article and supplementary materials.

## References

[1] Kim KH, Kabir E, Jahan SA. The use of personal hair dye and its implications for human health. Environ Int. 2016;89:222–7. 10.1016/j.envint.2016.01.018. 26895479 10.1016/j.envint.2016.01.018

[2] Hedberg YS, Uter W, Banerjee P, et al. Non-oxidative hair dye products on the European market: what do they contain? Contact Dermatitis. 2018;79(5):281–7. 10.1111/cod.13074. 30028011 10.1111/cod.13074

[3] Sayed SF, Dalai HG, Sharma M, Halawani R. Ecotoxicity, health risks and contact allergy due to p-phenylenediamine in hair dyes and tattoos: female students’ perspectives. Cureus. 2024;16(5):e60984. 10.7759/cureus.60984. 38910695 10.7759/cureus.60984 PMC11193909

[4] He L, Michailidou F, Gahlon HL, Zeng W. Hair dye ingredients and potential health risks from exposure to hair dyeing. Chem Res Toxicol. 2022;35(6):901–15. 10.1021/acs.chemrestox.1c00427. 35666914 10.1021/acs.chemrestox.1c00427 PMC9214764

[5] Bray F, Laversanne M, Sung H, et al. Global cancer statistics 2022: GLOBOCAN estimates of incidence and mortality worldwide for 36 cancers in 185 countries. CA Cancer J Clin. 2024;74(3):229–63. 10.3322/caac.21834. 38572751 10.3322/caac.21834

[6] Heikkinen S, Pitkäniemi J, Sarkeala T, Malila N, Koskenvuo M. Does hair dye use increase the risk of breast cancer? A population-based case-control study of Finnish women. PLoS ONE. 2015;10(8):e0135190. 10.1371/journal.pone.0135190. 26263013 10.1371/journal.pone.0135190 PMC4532449

[7] Eberle CE, Sandler DP, Taylor KW, White AJ. Hair dye and chemical straightener use and breast cancer risk in a large US population of black and white women. Int J Cancer. 2020;147(2):383–91. 10.1002/ijc.32738. 31797377 10.1002/ijc.32738 PMC7246134

[8] Liu C, Fan H, Li Y, Xiao X. Research advances on hepatotoxicity of herbal medicines in China. Biomed Res Int. 2016;2016:7150391. 10.1155/2016/7150391. 28078299 10.1155/2016/7150391 PMC5203888

[9] Yao W, Huo J, Ji J, Liu K, Tao P. Elucidating the role of gut microbiota metabolites in diabetes by employing network pharmacology. Mol med (Cambridge, Mass). 2024;30(1):263. 10.1186/s10020-024-01033-0. 39707185 10.1186/s10020-024-01033-0 PMC11660459

[10] Mu J, Li Y, Chen Q, et al. Revealing the molecular mechanism of baohuoside I for the treatment of breast cancer based on network pharmacology and molecular docking. J Ethnopharmacol. 2025;337(3):118918. 10.1016/j.jep.2024.118918. 39396715 10.1016/j.jep.2024.118918

[11] Sathish S, Sohn H, Madhavan T. An in silico approach to uncover selective JAK1 inhibitors for breast cancer from Life Chemicals Database. Appl Biochem Biotechnol. 2025;197(4):2508–43. 10.1007/s12010-024-05109-9. 39760987 10.1007/s12010-024-05109-9

[12] Sathish S, Devaraju P, Julius A, Sohn H, Madhavan T. Identification of selective inhibitors for Janus kinase 1: an integrated drug repurposing strategy for breast cancer. Chem Pap. 2024;78(1):245–62. 10.1007/s11696-023-03070-1.

[13] Jo S, Kim T, Iyer VG, Im W. CHARMM-GUI: a web-based graphical user interface for CHARMM. J Comput Chem. 2008;29(11):1859–65. 10.1002/jcc.20945. 18351591 10.1002/jcc.20945

[14] Zheng Y, Ji S, Li X, Feng Q. Active ingredients and molecular targets of *Taraxacum mongolicum* against hepatocellular carcinoma: network pharmacology, molecular docking, and molecular dynamics simulation analysis. PeerJ. 2022;10:e13737. 10.7717/peerj.13737. 35873910 10.7717/peerj.13737 PMC9302432

[15] Li C, Wen R, Liu D, Yan L, Gong Q, Yu H. Assessment of the potential of *Sarcandra glabra* (Thunb.) Nakai. in treating ethanol-induced gastric ulcer in rats based on metabolomics and network analysis. Front Pharmacol. 2022;13:810344. 10.3389/fphar.2022.810344. 35903344 10.3389/fphar.2022.810344 PMC9315220

[16] Rosenberg FM, Ofenloch RF, van der Most PJ, Snieder H, Schuttelaar MLA. Insights into hair dye use and self-reported adverse skin reactions in the Dutch general population: a cross-sectional questionnaire-based study. Contact Dermatitis. 2025;92(3):224–33. 10.1111/cod.14703. 39360575 10.1111/cod.14703 PMC11795336

[17] Piletta-Zanin A, Marchal O, Pastor D, Piletta-Zanin P, Harr T. An unusual case of a combined severe type I immediate hypersensitivity reaction and delayed type IV allergic contact dermatitis caused by hair dyes including toluene-2,5-diamine in the same patient. Contact Dermatitis. 2024;91(3):257–9. 10.1111/cod.14587. 38812256 10.1111/cod.14587

[18] Ishida W, Makino T, Shimizu T. Severe hair loss of the scalp due to a hair dye containing para phenylenediamine. ISRN Dermatol. 2011;2011:947284. 10.5402/2011/947284. 22363863 10.5402/2011/947284 PMC3262542

[19] Helaskoski E, Suojalehto H, Virtanen H, et al. Occupational asthma, rhinitis, and contact urticaria caused by oxidative hair dyes in hairdressers. Ann Allergy Asthma Immunol. 2014;112(1):46–52. 10.1016/j.anai.2013.10.002. 24331393 10.1016/j.anai.2013.10.002

[20] Gago-Dominguez M, Castelao JE, Yuan JM, Yu MC, Ross RK. Use of permanent hair dyes and bladder-cancer risk. Int J Cancer. 2001;91(4):575–9. 11251984 10.1002/1097-0215(200002)9999:9999<::aid-ijc1092>3.0.co;2-s

[21] Qin L, Deng HY, Chen SJ, Wei W. A meta-analysis on the relationship between hair dye and the incidence of Non-Hodgkin’s lymphoma. Med Princ Pract. 2019;28(3):222–30. 10.1159/000496447. 30583293 10.1159/000496447 PMC6597908

[22] Xu S, Wang H, Liu Y, et al. Hair chemicals may increase breast cancer risk: a meta-analysis of 210319 subjects from 14 studies. PLoS ONE. 2021;16(2):e0243792. 10.1371/journal.pone.0243792. 33539348 10.1371/journal.pone.0243792 PMC7861401

[23] McGregor DB, Brown A, Cattanach P, et al. Responses of the L5178Y tk+/tk- mouse lymphoma cell forward mutation assay: III. 72 coded chemicals. Environ Mutagen. 1988;12(1):85–154. 10.1002/em.2860120111. 10.1002/em.2860120111 3383842

[24] Lin T, Qiu Y, Peng W, Peng L. Heat shock protein 90 family isoforms as prognostic biomarkers and their correlations with immune infiltration in breast cancer. Biomed Res Int. 2020;2020:2148253. 10.1155/2020/2148253. 33145341 10.1155/2020/2148253 PMC7596464

[25] Peng C, Zhao F, Li H, Li L, Yang Y, Liu F. HSP90 mediates the connection of multiple programmed cell death in diseases. Cell Death Dis. 2022;13(11):929. 10.1038/s41419-022-05373-9. 36335088 10.1038/s41419-022-05373-9 PMC9637177

[26] Zhang Y, Zhao G, Yu L, et al. Heat-shock protein 90α protects NME1 against degradation and suppresses metastasis of breast cancer. Br J Cancer. 2023;129(10):1679–91. 10.1038/s41416-023-02435-3. 37731021 10.1038/s41416-023-02435-3 PMC10645775

[27] Han Z, Jia Q, Zhang J, et al. Deubiquitylase YOD1 regulates CDK1 stability and drives triple-negative breast cancer tumorigenesis. J Exp Clin Cancer Res. 2023;42(1):228. 10.1186/s13046-023-02781-3. 37667382 10.1186/s13046-023-02781-3 PMC10478497

[28] Gupta J, Saeed BI, Bishoyi AK, et al. From cell cycle control to cancer therapy: exploring the role of CDK1 and CDK2 in tumorigenesis. Med Oncol. 2025;42(9):422. 10.1007/s12032-025-02973-1. 40782258 10.1007/s12032-025-02973-1

[29] Song L, Liu Z, Hu HH, et al. Proto-oncogene Src links lipogenesis via lipin-1 to breast cancer malignancy. Nat Commun. 2020;11(1):5842. 10.1038/s41467-020-19694-w. 33203880 10.1038/s41467-020-19694-w PMC7672079

[30] Finn RS. Targeting Src in breast cancer. Ann Oncol. 2008;19(8):1379–86. 10.1093/annonc/mdn291. 18487549 10.1093/annonc/mdn291

